# Replication of an Autonomous Human Parvovirus in Non-dividing Human Airway Epithelium Is Facilitated through the DNA Damage and Repair Pathways

**DOI:** 10.1371/journal.ppat.1005399

**Published:** 2016-01-14

**Authors:** Xuefeng Deng, Ziying Yan, Fang Cheng, John F. Engelhardt, Jianming Qiu

**Affiliations:** 1 Department of Microbiology, Molecular Genetics and Immunology, University of Kansas Medical Center, Kansas City, Kansas, United States of America; 2 Department of Anatomy and Cell Biology, College of Medicine, University of Iowa, Iowa City, Iowa, United States of America; University of North Carolina at Chapel Hill, UNITED STATES

## Abstract

Human bocavirus 1 (HBoV1) belongs to the genus *Bocaparvovirus* of the *Parvoviridae* family, and is an emerging human pathogenic respiratory virus. In vitro, HBoV1 infects well-differentiated/polarized primary human airway epithelium (HAE) cultured at an air-liquid interface (HAE-ALI). Although it is well known that autonomous parvovirus replication depends on the S phase of the host cells, we demonstrate here that the HBoV1 genome amplifies efficiently in mitotically quiescent airway epithelial cells of HAE-ALI cultures. Analysis of HBoV1 DNA in infected HAE-ALI revealed that HBoV1 amplifies its ssDNA genome following a typical parvovirus rolling-hairpin DNA replication mechanism. Notably, HBoV1 infection of HAE-ALI initiates a DNA damage response (DDR) with activation of all three phosphatidylinositol 3-kinase–related kinases (PI3KKs). We found that the activation of the three PI3KKs is required for HBoV1 genome amplification; and, more importantly, we identified that two Y-family DNA polymerases, Pol η and Pol κ, are involved in HBoV1 genome amplification. Overall, we have provided an example of *de novo* DNA synthesis (genome amplification) of an autonomous parvovirus in non-dividing cells, which is dependent on the cellular DNA damage and repair pathways.

## Author Summary

Parvovirus is unique among DNA viruses. It has a single stranded DNA genome of ~5.5 kb in length. Autonomous parvoviruses, which replicate autonomously in cells, rely on the S phase cell cycle for genome amplification. In the current study, we demonstrated that human bocavirus 1 (HBoV1), an autonomous human *Bocaparvovirus*, replicates its genome in well-differentiated (non-dividing) primary human airway epithelial cells. HBoV1 infection of non-dividing human airway epithelial cells induces a DNA damage response. We provide evidence that HBoV1 genome amplification in non-dividing airway epithelial cells is facilitated by the DNA damage response-mediated signaling pathways. Importantly, we discovered that two Y-family DNA repair polymerases, but not cellular DNA replication polymerases, are directly involved in HBoV1 genome amplification. Therefore, our study is innovative because it is the first to show that an autonomous parvovirus amplifies its genome in non-dividing cells, and that the DNA repair polymerases are involved in viral genome amplification.

## Introduction

Human bocavirus 1 (HBoV1) belongs to the *Bocaparvovirus* genus in the *Parvoviridae* family [1,2]. HBoV1 is one of a group of etiological respiratory viruses that cause acute respiratory tract infections in young children. Wheezing is one of the most common symptoms of the virus infection [3,4]. Acute HBoV1 infection, diagnosed by detection of HBoV1-specific IgM/an increased HBoV1-specific IgG antibody in serum, a virus load higher than 1 × 10^4^ viral genome copy numbers (gc)/ml, or HBoV1 mRNA in nasopharyngeal aspirates, or diagnosed HBoV1 viremia, results in respiratory illness [3,5–10]. Life-threatening HBoV1 infections in pediatric patients have been reported [11].

Studies of children with pneumonia, acute wheezing, asthma, and/or bronchiolitis suggest that HBoV1 infects the lower respiratory airways down to the bronchioles [3,5]. In vitro, HBoV1 infects well-differentiated or polarized human primary airway epithelium (HAE) cultured at an air-liquid interface (HAE-ALI) [12]. The in vitro model of HAE-ALI, which is derived from primary human bronchial epithelial cells, is a novel system that has provided new insights into the infection characteristics of human respiratory RNA viruses [13,14], as well as respiratory DNA viruses [15]. We have demonstrated that HBoV1 infection of HAE-ALI is long-lasting, persistent, and productive, causing a remarkable loss of epithelial integrity [16,17], which is consistent with the prolonged primary shedding events of HBoV1 for up to a year in patients with respiratory illness [18].

In general, autonomous parvovirus replication is dependent on the S phase of the infected cells because the incoming single-stranded genome of the parvovirus does not support transcription and relies on the host cell DNA replication machinery [19–22]. Except for HBoV1 infection of HAE-ALI, there have been no reports to date of productive infection or viral DNA replication of autonomous parvoviruses in mitotically quiescent cells. *Dependoparvovirus* adeno-associated virus (AAV) of the *Parvoviridae* family, on the other hand, depends on a helper virus, e.g., adenovirus or herpes simplex virus, or DNA damaging agents [23], for its genome replication. These helper viruses induce a cellular environment conducive to AAV replication. AAV DNA replication has been studied extensively in culture of dividing cells; however, how AAV replicates in the context of the non-dividing cells of the host remains elusive [23].

In this report, we studied the mechanism underlying genome amplification of human parvovirus HBoV1 in well-differentiated (non-dividing) airway epithelial cells of the HAE-ALI culture. We demonstrated that HBoV1 infection of HAE-ALI induces a DNA damage response (DDR) that facilitates viral genome amplification. Importantly, we provide evidence that Y-family DNA repair polymerases, Pol η and Pol κ, are involved in HBoV1 genome amplification. To our knowledge, this is the first report to show that parvovirus DNA replicates in non-dividing cells autonomously.

## Results

### HBoV1 genome amplification in non-dividing human airway epithelial cells

**Fig 1:**
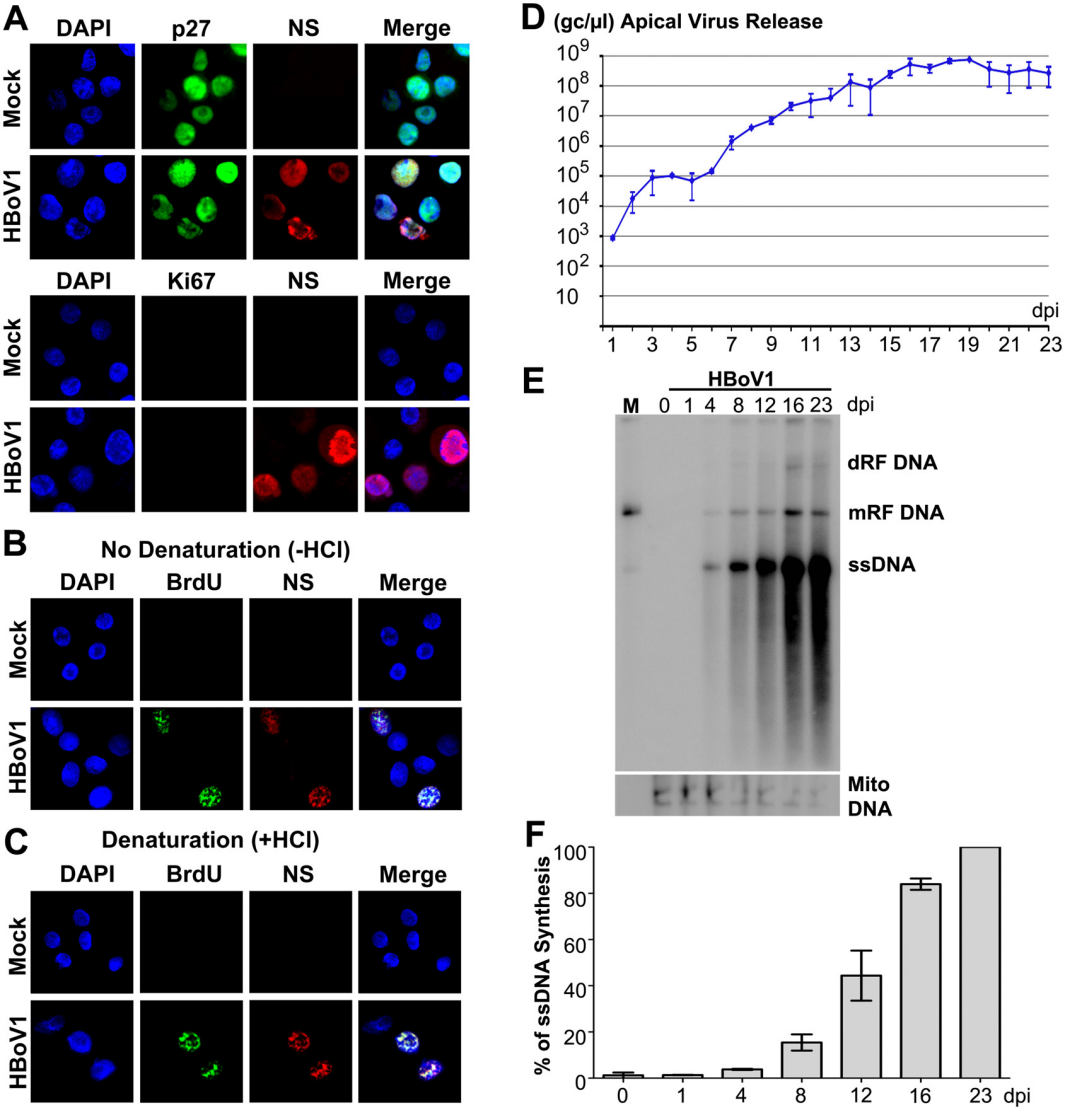
HBoV1 replicates in non-dividing airway epithelial cells of HAE-ALI cultures. (**A**) HBoV1 infection of non-dividing cells. At 12 dpi, both mock- and HBoV1-infected cells of HAE-ALI cultures were trypsinized off the inserts, cytospun onto a slide, and analyzed by IF with anti-HBoV1 NS1C and anti-p27 antibodies, and with anti-NS1C and anti-Ki67 antibodies, respectively. (**B** and **C**) Detection of HBoV1 DNA replication by BrdU incorporation assay. At 12 dpi, mock- and HBoV1-infected cells of the HAE-ALI cultures were trypsinized off the inserts, and were labeled with BrdU. The labeled cells were then cytospun onto a slide, treated without (**B**) or with HCl (**C**), as indicated. The cells were co-stained with anti-BrdU and anti-NS1C. Nuclei were stained with DAPI (blue), and the cells were visualized by confocal microscopy at a magnification of ×100. (**D**) Quantification of apical virus release. At the indicated dpi, the apical surface was washed with 100 μl of PBS to collect the released virus. DNase I digestion-resistant HBoV1 genome copy numbers were quantified by qPCR (Y-axis) and plotted to the dpi as shown. Means and standard deviations from three independent experiments (n = 3) are shown. (E&F) Analysis of viral DNA replication by Southern blotting. (**E**) At the indicated dpi, Hirt DNA samples isolated from infected-HAE-ALI cultures were analyzed by Southern blotting with a probe spanning the HBoV1 *NS* and *Cap* genes (upper panel), and with a probe specifically used to detect mitochondrial DNA (Mito DNA; lower panel), respectively. dRF DNA, double replicative form (RF) DNA; mRF DNA, monomer RF DNA; ssDNA, single stranded DNA. An HBoV1 RF DNA (M), which was digested from pIHBoV1, was used as a marker (5.4 kb). (**F**) The level of viral ssDNA detected in the blot was quantified and normalized to the Mito DNA detected in the same sample, and the % of the viral ssDNA relative to that at 23 dpi is shown. Averages and standard deviations (n = 3) were shown.

We examined the cell cycle status of HBoV1-infected cells of HAE-ALI. We used polarized HAE-ALI cultures that had a transepithelial electrical resistance (TEER) of >1.5 KΩ for infection. We found that the HAE cells of the ALI cultures were well differentiated with p27 expression, which is a marker of G0 phase [24], but without expression of proliferating cell nuclear antigen (PCNA), which is a marker of cellular DNA replication [25], or expression of Ki67, which marks all phases of the cell cycle including S phase [26]. (S1A, S1B and S1C Fig). Therefore, polarized HAE-ALI cultures are largely composed of non-dividing cells. HBoV1 infected p27-expressing cells, as shown by co-immunostaining of anti-p27 and anti-HBoV1 NS1C antibodies (Fig 1A, p27). The anti-NS1C antibody recognizes both the large and small viral non-structural proteins (NS) expressed during HBoV1 infection [27]. HBoV1-infected cells also did not express Ki67 (Fig 1A, Ki67). Proliferating primary human airway epithelial cells in monolayer culture, for which over half of the cells are proliferating in S phase and do not support HBoV1 DNA replication [16], were used as negative and positive controls for staining with anti-p27 and anti-Ki67, respectively, and were not infected by HBoV1 (S1D Fig).

We next used a BrdU incorporation assay to pulse-chase viral genome amplification. In this assay, denaturation is necessary to detect the BrdU incorporated in double-stranded DNA (dsDNA), but not single-stranded DNA (ssDNA) [28]. In the absence of HCl treatment (no denaturation), NS-expressing cells incorporated BrdU into viral ssDNA, as shown by co-immunostaining of the anti-NS1C and anti-BrdU antibodies (Fig 1B), indicating viral ssDNA synthesis. Notably, under the denaturation condition, mock-infected HAE cells did not incorporate BrdU (Fig 1C), confirming that there was no obvious cellular DNA synthesis in HAE cells as also supported by the lack of Ki67 staining (Fig 1A), which marks all phases of the cell cycle.

In infected HAE-ALI, HBoV1 virions were released daily from the apical side, and reached a level of > 10^10^ gc/well at 16–23 days post-infection (dpi) (Fig 1D, 10^8^ gc/μl). Viral ssDNA genome amplification in infected HAE-ALI was confirmed (Fig 1E and 1F), which undergoes intermediates of double and mono replicative forms (dRF and mRF, respectively), a procedure similar to the DNA replication of minute virus of mice (MVM), an autonomous parvovirus [29]. We observed a roughly linear increase in the ssDNA synthesis vs. a several log increase in progeny virion release over time (Fig 1D and 1E). We speculate that the synthesized viral ssDNA genomes are rapidly packaged into capsids, and the matured virions are rapidly released from the cells.

Collectively, these results confirmed that HBoV1 amplifies its ssDNA genome in non-dividing airway epithelial cells of the HAE-AL culture and produces progeny virions over the course of infection.

### HBoV1 infection of non-dividing airway epithelial cells induces a DNA damage response (DDR)

**Fig 2:**
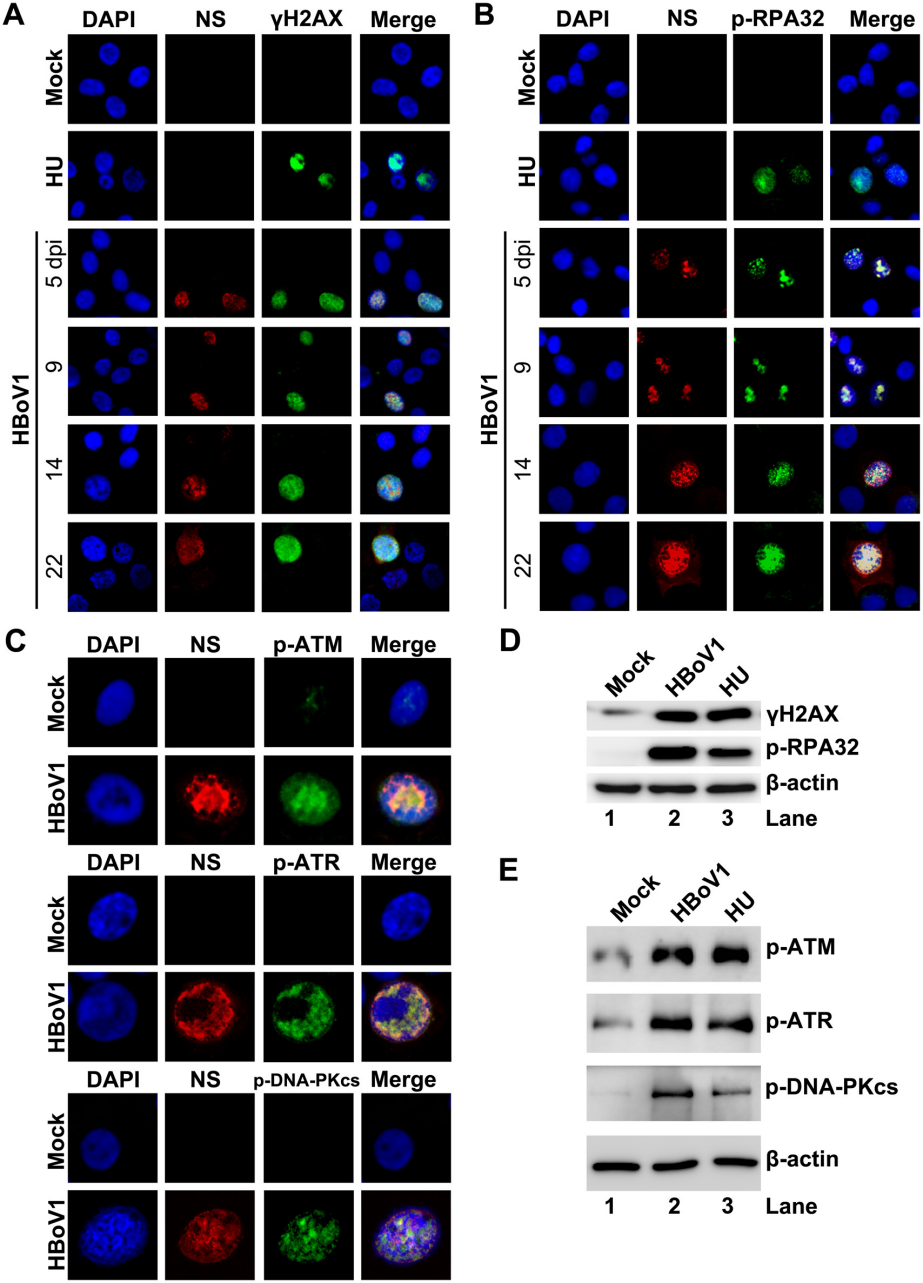
HBoV1 infection of HAE-ALI cultures induces phosphorylation of H2AX and RPA32 and activates ATM, ATR, and DNA-PKcs. HAE-ALI cultures were infected with HBoV1 or mock-infected. (**A**, **B**, and C) IF analysis. (**A** and **B**) At the indicated dpi, the infected cells trypsinized off the insert were cytospun and co-immunostained with anti-NS1C and anti-γH2AX (**A**), and with anti-NS1C and anti-p-RPA32 (**B**). (**C**) At 10 dpi, infected cells trypsinized off the ALI membrane were used for IF analysis with anti-NS1C and p-ATM, with anti-NS1C and anti-p-ATR, and with anti-NS1C and anti-p-DNA-PKcs, as indicated. Nuclei were stained with DAPI (blue), and the cells were visualized by confocal microscopy at a magnification of × 100. (**D** and **E**) Western-blot analysis. At 10 dpi, cells of the mock-, HBoV1-infected, or HU-treated HAE-ALI cultures were lysed in 1 × SDS-containing loading buffer. Equivalent volumes of the lysate were used for Western blot using anti-γH2AX, and reprobed with p-RPA32 and anti-β-actin, sequentially (**D**), and using with anti-p-ATM, anti-p-ATR, anti-p-DNA-PKcs, and anti-β-actin, respectively (**E**). HAE-ALI cultures treated with HU at a final concentration of 2 mM for 2 days were used as positive control.

Key factors of DNA replication, such as proliferating cell nuclear antigen (PCNA) and DNA polymerase (Pol) δ, are not expressed in non-dividing cells [30]; therefore, how HBoV1 genome amplifies in infected HAE-ALI without these proteins remains an enigma. We therefore looked into the DNA damage and repair pathways in HBoV1-infected HAE cells. In NS-expressing cells, both RPA32 (replication protein A 32) and histone variant H2AX (H2A histone family, member X) were phosphorylated, as detected using antibodies against p-RPA32 (the RPA32 phosphorylated on serine 33) and γH2AX (the H2AX phosphorylated on serine 139), over the course of infection (Fig 2A and 2B), suggesting that HBoV1 infection induces a DDR. It is thought that three phosphatidylinositol 3-kinase-like kinases (PI3KKs) are responsible for the DDR [31,32], we next looked at the activation status of the three PI3KKs. We found that all three PI3KKs, ATM (Ataxia telangiectasia mutated), ATR (ATM- and RAD3-related), and DNA-PKcs (DNA-dependent protein kinase catalytic subunit), were activated in infected cells and colocalized with NS, as assessed by immunofluorescence (IF) analysis using antibodies against the specifically phosphorylated site of each kinase (Fig 2C). Phosphorylation of RPA32, H2AX, ATM, ATR, and DNA-PKcs was also confirmed by Western blotting (Fig 2D and 2E). ATM, ATR and DNA-PKcs are phosphorylated at serine 1981, threonine 1989, and serine 2056, respectively, which are all functional phosphorylation sites that are required for DDR signaling [33–35]. As a control, treatment with hydroxyurea (HU) also induced phosphorylation of these proteins (Fig 2D and 2E).

### Inhibition of ATM, ATR, or DNA-PKcs phosphorylation significantly decreases HBoV1 genome amplification

**Fig 3:**
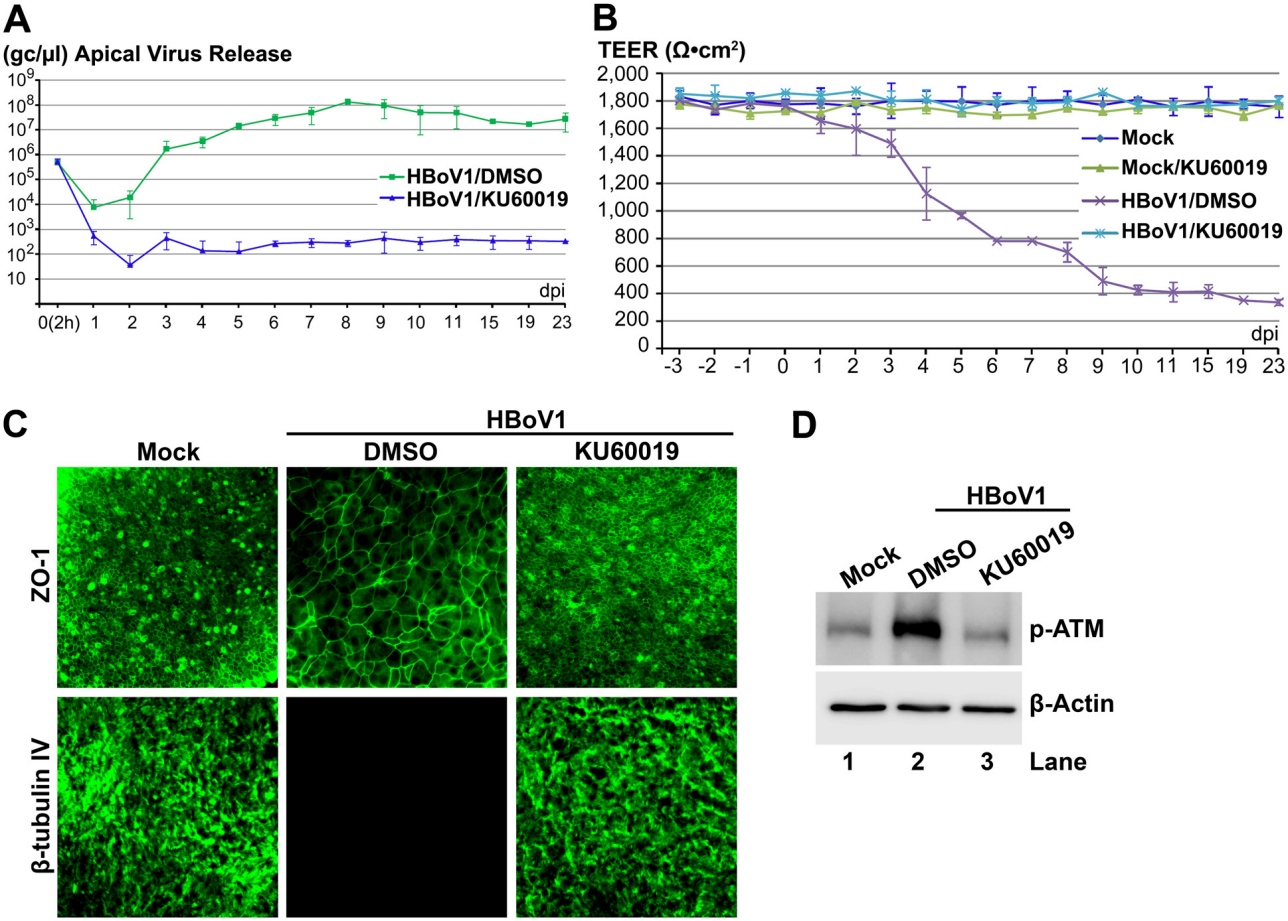
An ATM-specific inhibitor decreases HBoV1 infection of HAE-ALI. Two days prior to apical infection of HBoV1, HAE-ALI cultures were treated with KU60019 at a final concentration of 40 μM in the basolateral chamber, which was refreshed every three days along with the ALI medium in the basolateral chamber. (**A**) Quantification of apical virus release. At the indicated dpi, apical washes were collected and quantified for HBoV1 genome copy numbers (Y-axis), which are plotted to the dpi as shown. Averages and standard deviations (n = 3) are shown. (**B**) TEER measurement. At the indicated dpi, the TEER of drug-treated mock-/HBoV1-infected HAE-ALI cultures, as indicated, was measured. Means and standard deviations (n = 3) are shown. (**C**) IF analysis. At 23 dpi, the ALI membrane of the infected HAE-ALI cultures was stained with anti-β-tubulin IV or with anti-ZO-1, as indicated. The stained membranes were visualized for β-tubulin IV/ZO-1 (green) expression by confocal microscopy at a magnification of × 40. (**D**) Analysis of phosphorylated ATM. At 23 dpi, equivalent cells of the infected HAE-ALI cultures were analyzed by Western blot for expression of p-ATM and β-actin, respectively.

To functionally interrogate the requirement for PI3KK activation in mediating viral genome amplification, we applied ATM-, ATR-, or DNA-PKcs-pharmacological inhibitors, which specially inhibit phosphorylation of their respective kinases, to HAE-ALI cultures and evaluated their effects on viral genomes released from the apical surface. Application of an ATM-specific inhibitor, KU60019 [36], at a concentration of 40 μM, decreased apical virion release by 4–5 log starting at 4 dpi (Fig 3A). Application of the KU60019 also prevented infection-dependent barrier dysfunction, as demonstrated by the lack of a decline in TEER (Fig 3B), no significant dissociation of the tight junction protein Zona occludens-1 (ZO-1) [37], and no loss of cilia (β-tubulin IV expression) (Fig 3C), which were all observed in the vehicle (DMSO) treated HBoV1-infected group (Fig 3B and 3C). Application of KU60019 also effectively reduced phosphorylation of ATM in HBoV1-infected HAE-ALI cultures to a level observed in mock-infected cells (Fig 3D).

**Fig 4:**
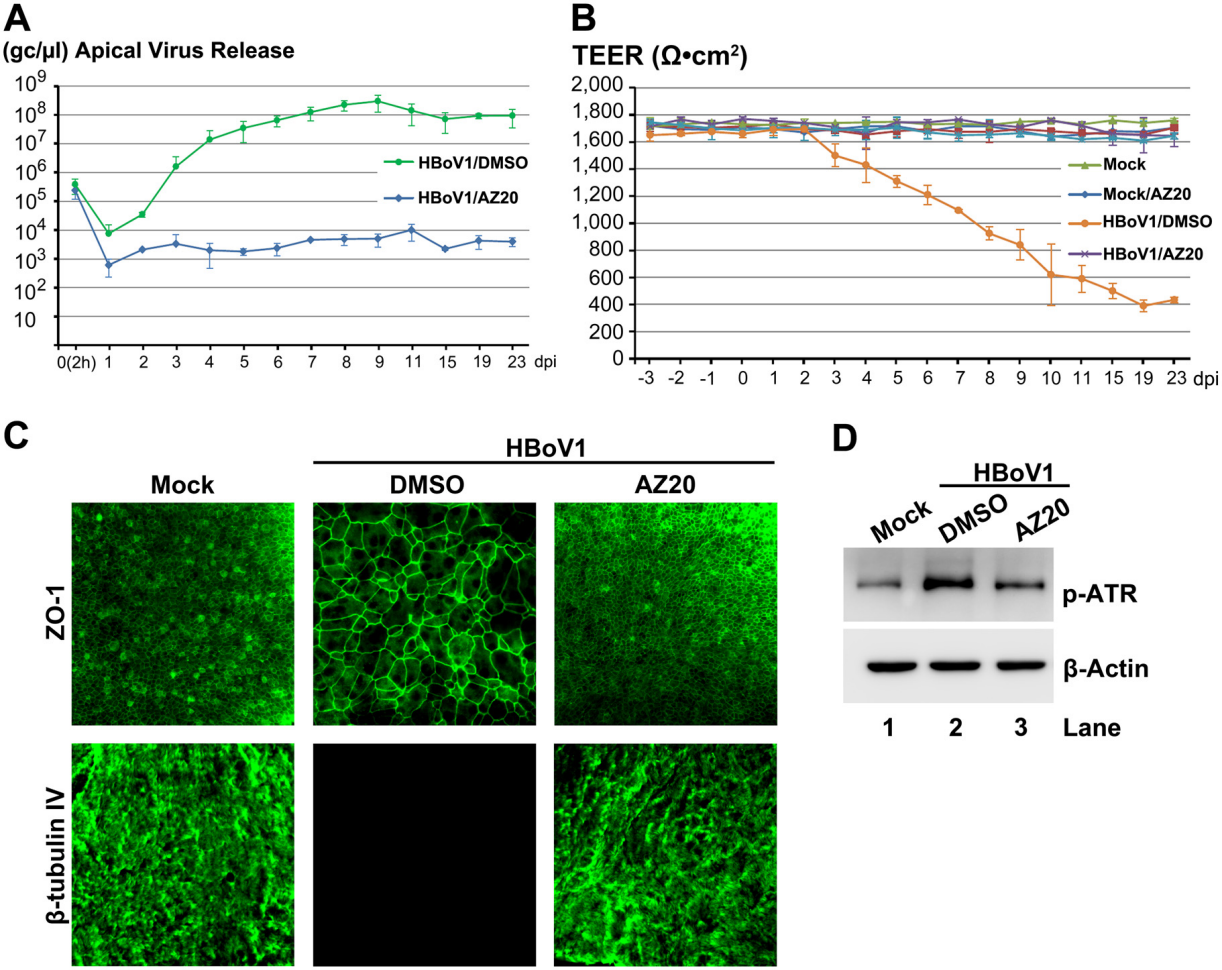
An ATR-specific inhibitor decreases HBoV1 infection of HAE-ALI. At two days prior to infection, HAE-ALI cultures were treated with AZ20 at 20 μM from the basolateral side. The treated cultures were then infected with HBoV1. (**A**) Quantification of apical virus release. At the indicated dpi, the apical washes were quantified for HBoV1 genome copies by qPCR (Y-axis) and plotted to the dpi as shown. Means and standard deviations (n = 3) are shown. (**B**) TEER measurement. At the indicated dpi, the TEER of infected HAE-ALI cultures, as indicated, was measured. Means and standard deviations (n = 3) are shown. (**C**) IF analysis. At 23 dpi, the ALI membrane of the infected HAE-ALI cultures were stained with anti-β-tubulin IV or with anti-ZO-1, as indicated. The stained membranes were visualized for β-tubulin IV/ZO-1 (green) expression by confocal microscopy at a magnification of × 40. (**D**) Analysis of phosphorylated ATR. At 23 dpi, equivalent cells of the infected HAE-ALI cultures were analyzed by Western blot for expression of p-ATR and β-actin, respectively.

**Fig 5:**
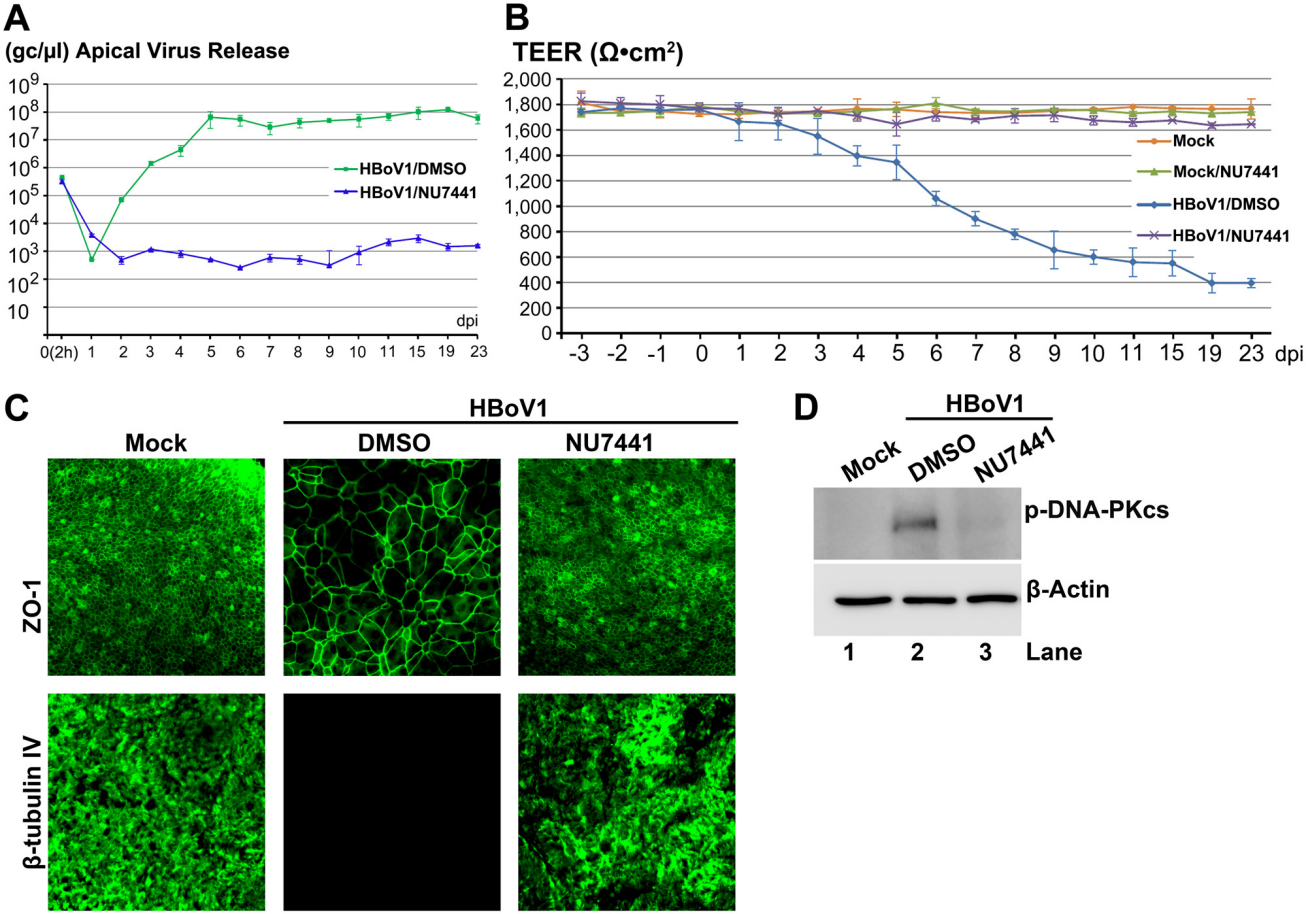
A DNA-PKcs-specific inhibitor decreases HBoV1 infection of HAE-ALI. At two days prior to apical infection of HBoV1, HAE-ALI cultures were incubated with NU7441 at 20 μM in the basolateral chamber. (**A**) Quantification of apical virus release. At the indicated dpi, apical washes were quantified for HBoV1 genome copies qPCR (Y-axis) and plotted to the dpi as shown. Means and standard deviations (n = 3) are shown. (**B**) TEER measurement. At the indicated dpi, the TEER of infected HAE-ALI cultures, as indicated, was determined. Means and standard deviations (n = 3) are shown. (**C**) IF analysis. At 23 dpi, the ALI membrane of the infected HAE-ALI cultures was stained with anti-β-tubulin IV or with anti-ZO-1, as indicated. The stained membranes were visualized for β-tubulin IV/ZO-1 (green) expression by confocal microscopy at a magnification of × 40. (**D**) Analysis of phosphorylated DNA-PKcs. At 23 dpi, equivalent cells of the infected HAE-ALI cultures were analyzed by Western blotting for expression of p-DNA-PKcs and β-actin, respectively.

Similarly, we examined an ATR-specific inhibitor AZ20 [38]. At 20 μM, AZ20 inhibited apical virus release by 4 log over the course of 6–23 dpi (Fig 4A), and prevented airway epithelial damage, which was marked by disruption of the TEER (Fig 4B) and the dissociation of ZO-1 and no expression of β-tubulin IV (Fig 4C), which were observed in the vehicle-treated HBoV1 infected group (Fig 4B and 4C). Application of AZ20 reduced ATR phosphorylation of HBoV1-infected cells to near background levels observed in mock-infected cells (Fig 4D). Inhibition of apical virus release using the DNA-PKcs-specific inhibitor NU7441 [39] was also substantial and gave results strikingly similar to that of KU60019. At a concentration of 20 μM, NU7441 decreased apical virion release by 4–5 log over a period of 5–23 dpi (Fig 5A), and prevented the epithelial barrier damage caused by virus infection (Fig 5B and 5C). Applying NU7441 nearly abolished DNA-PKcs phosphorylation in HBoV1-infected cells (Fig 5D).

Applying KU60019, AZ20 and NU7441 alone at the concentrations used did not alter epithelial barrier function. The TEER remained >1.6K Ω (Figs 3B, 4B and 5B, compare Mock/KU, AZ or NU with Mock), and cell viability, which was assessed by cellular ATPase activity, was unchanged (S2 Fig). However, the three inhibitors reduced the phosphorylation of their respective kinases in HBoV1-infected cells to a background level of mock-infected cells (Figs 3D, 4D and 5D). Taken together, these results demonstrate that the HBoV1 infection-dependent phosphorylation of ATR, ATM, and DNA-PKcs is critical for HBoV1 genome amplification.

### Knockdown of ATM, ATR or DNA-PKcs significantly decreases HBoV1 genome amplification

**Fig 6:**
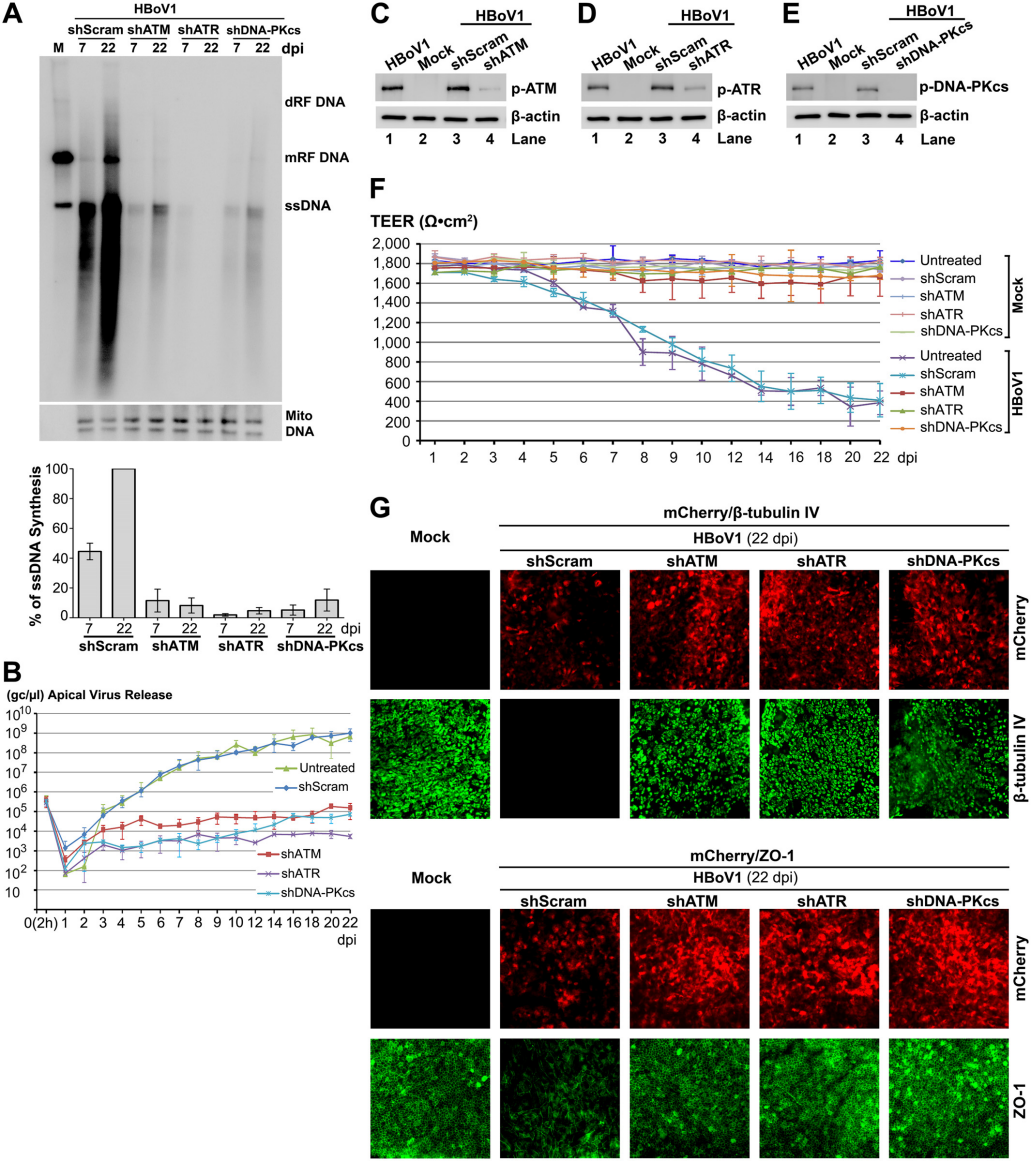
ATM-, ATR-, and DNA-PKcs-specific shRNAs inhibit HBoV1 DNA replication and attenuate the epithelial damage caused by HBoV1 infection. The HAE-ALI cultures, which expressed shScram, shATM, shATR, and shDNA-PKcs, as indicated, were infected with HBoV1. (**A**) Southern blot analysis of viral DNA replication. At the indicated dpi, Hirt DNA was extracted from infected cultures and analyzed by Southern blotting using the HBoV1 *NSCap* probe (upper) and the mitochondrial DNA probe (lower). A representative blot is shown. The level of viral ssDNA detected in the blot was further quantified and normalized to the Mito DNA detected in the same sample. The % of the viral ssDNA relative to that of the shScram-applied sample at 22 dpi is shown. Averages and standard deviations (n = 3) were shown at the bottom. (**B**) Quantification of apical virus release. At the indicated dpi, apical washes were quantified for HBoV1 genome copy numbers by qPCR (Y-axis) and plotted to the dpi as shown. Means and standard deviations (n = 3) are shown. (**C**, **D**, and **E**) Western blot analysis of p-ATM, p-ATR, and p-DNA-PKcs expression. At 22 dpi, cells in the infected HAE-ALI cultures were analyzed by Western blotting for expression of p-ATM (**C**), p-ATR (**D**) and p-DNA-PKcs (**E**). β-actin was probed as a loading control. (**F**) TEER measurement. At the indicated dpi, the TEER of infected HAE-ALI cultures, as indicated, was measured. Means and standard deviations (n = 3) are shown. (**G**) IF analysis. At 22 dpi, mock-infected and HBoV1-infected HAE-ALI cultures transduced with various shRNA/mCherry-expressing lentiviruses, as indicated, were stained with anti-β-tubulin IV or with anti-ZO-1. The stained membranes were visualized for β-tubulin IV/ZO-1 (green) and mCherry (red) expression by confocal microscopy at a magnification of × 40.

To confirm the function of the three PI3KKs in HBoV1 genome amplification, we used ATR-, ATM-, or DNA-PKcs-specific shRNA. We generated lentiviral vectors that co-expressed each shRNA with an mCherry reporter to transduce monolayer cultures of proliferating airway epithelial cells, prior to seeding for ALI cultures. Stable and efficient transduction was evidenced by the expression of mCherry reporter in virtually all the cells of well-differentiated ALI cultures at 4 weeks post-transduction (S3A Fig). At this time, the ATM-, ATR-, and DNA-PKcs-specific shRNA-expressing HAE-ALI cultures demonstrated decreased expression of ATM, ATR and DNA-PKsc, respectively (by >4-fold), but the reduction of PI3KK expression was not observed in the shScram-expressing HAE-ALI (S3B Fig). We then infected these shRNA-expressing ALI cultures with HBoV1, and analyzed viral DNA replication in them. Viral DNA of either the mRF or ssDNA form in shATM-, shATR-, and shDNA-PKcs-expressing ALI cultures decreased dramatically at both 7 and 22 days, compared to those in shScram-expressing cultures (Fig 6A). Correspondently, apical virus release decreased by 3–4 log from 7 to 22 dpi, in shATM, shATR, and shDNA-PKcs-expressing HAE-ALI, but not in shScram-expressing HAE-ALI (Fig 6B). At 22 dpi, significantly decreased phosphorylation of ATM, ATR and DNA-PKcs was confirmed in their respective shRNA-expressing HAE-ALI, but the HBoV1 infection-dependent phosphorylation in shScram-expressing HAE-ALI remained at the same level as high as that in the HBoV1-infected HAE-ALI (Fig 6C, 6D and 6E). In response to the reduced HBoV1 infection, the shATM, shATR, and shDNA-PKcs-expressing HAE-ALI showed neither a significant decrease in TEER (Fig 6F), nor an obvious dissociation of the tight junction protein ZO-1, nor a total loss of β-tubulin IV-expressing cilia, which were otherwise observed in infected shScram-applied HAE-ALI (Fig 6G). As controls, shRNA expression alone did not affect the barrier function (TEER) (Fig 6F).

Taken together, the above results confirmed that all three PI3KKs (ATM, ATR and DNA-PKs) play an important role in HBoV1 genome amplification in HAE-ALI.

### DNA repair polymerases are involved in HBoV1 genome amplification in HAE-ALI

**Fig 7:**
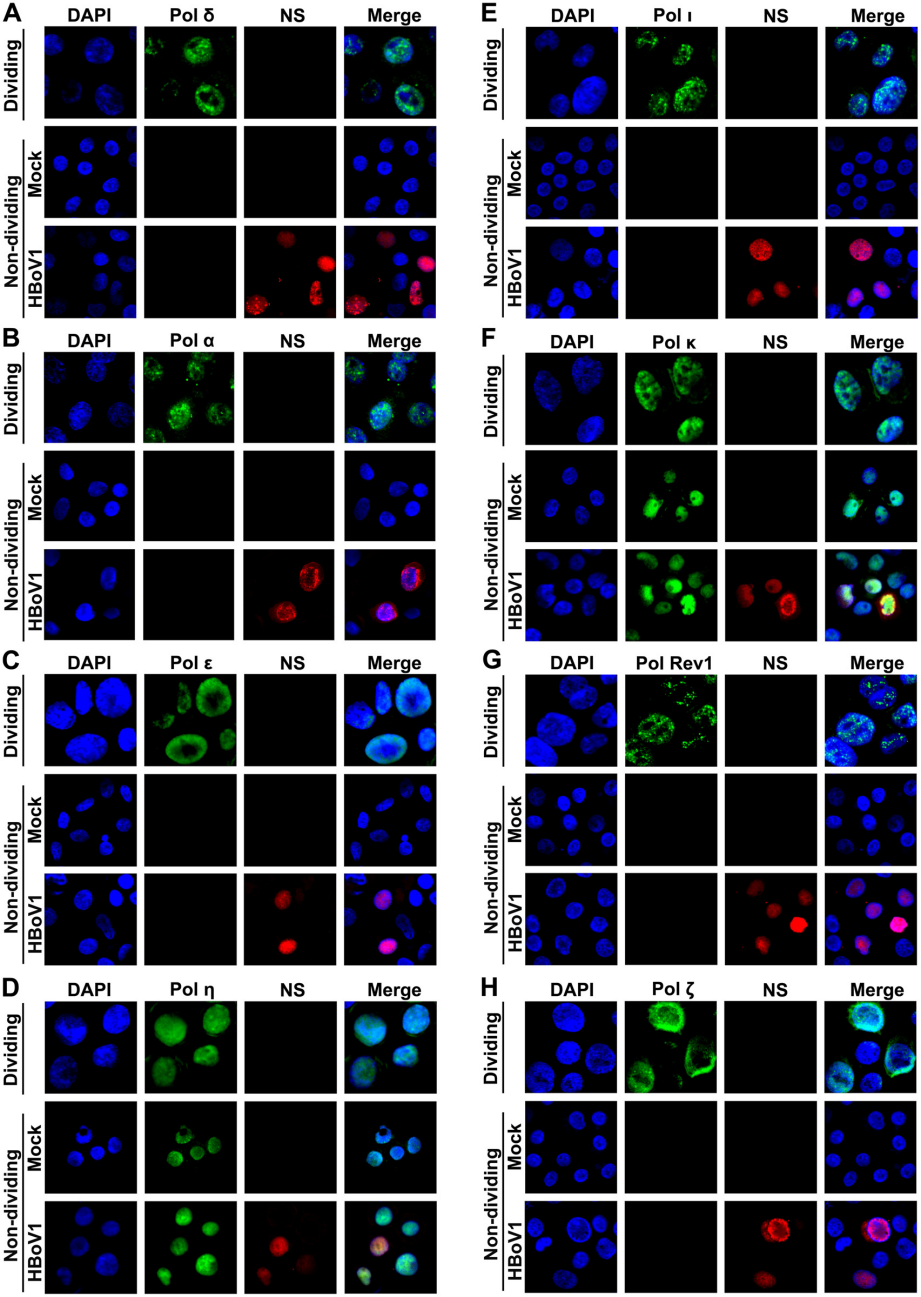
IF analysis of DNA polymerases, Pol δ, Pol α, Pol ε, Pol η, Pol ι, Pol κ, REV1, and Pol ζ, in the cells of HAE-ALI. HAE-ALI cultures, labeled as “Non-dividing,” were infected with HBoV1 or mock-infected. At 12 dpi, HAE cells trypsinized off the Transwell inserts were analyzed by IF using anti-Pol δ (**A**), anti-Pol α (**B**), anti-Pol ε (**C**), anti-Pol η (**D**), anti-Pol ι (**E**), anti-Pol κ (**F**), anti-Pol Rev1 (**G**), and anti-Pol ζ (**H**) antibodies. Primary airway epithelial cells, labeled as “Dividing,” cultured as monolayer in a flask were stained with the above antibodies as antibody positive control. Nuclei were stained with DAPI (blue), and the stained cells were visualized by confocal microscopy at a magnification of ×100.

**Fig 8:**
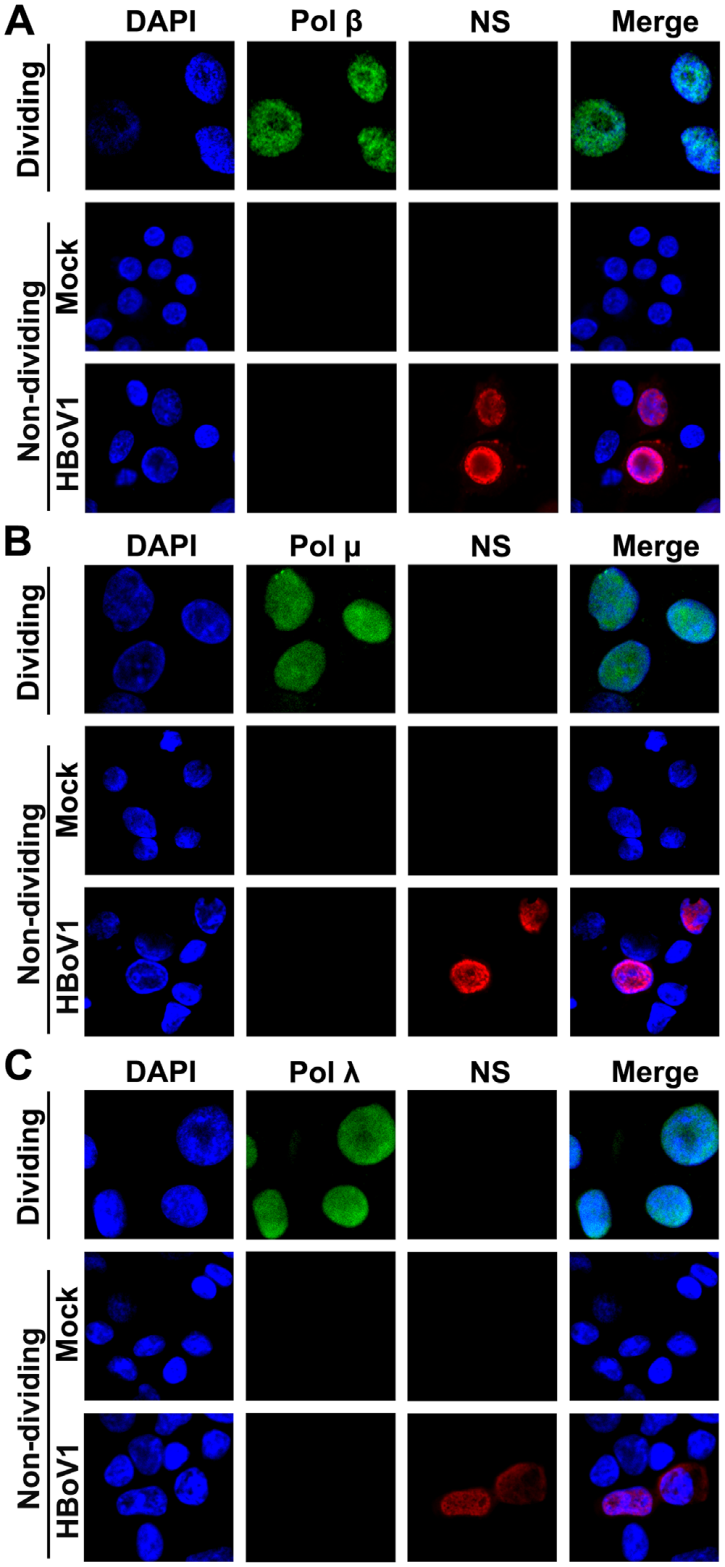
IF analysis of DNA polymerases, Pol β, Pol μ, and Pol λ, in the cells of HAE-ALI. HAE-ALI cultures, labeled as “Non-dividing,” were infected with HBoV1 or mock-infected. At 12 dpi, HAE cells trypsinized off the Transwell inserts were analyzed by IF using anti-Pol β (**A**), anti-Pol μ (**B**), and anti-Pol λ (**C**) antibodies. Primary airway epithelial cells, labeled as “Dividing,” cultured as monolayer in a flask were stained with the above antibodies as antibody positive control. Nuclei were stained with DAPI (blue), and the stained cells were visualized by confocal microscopy at a magnification of ×100.

**Fig 9:**
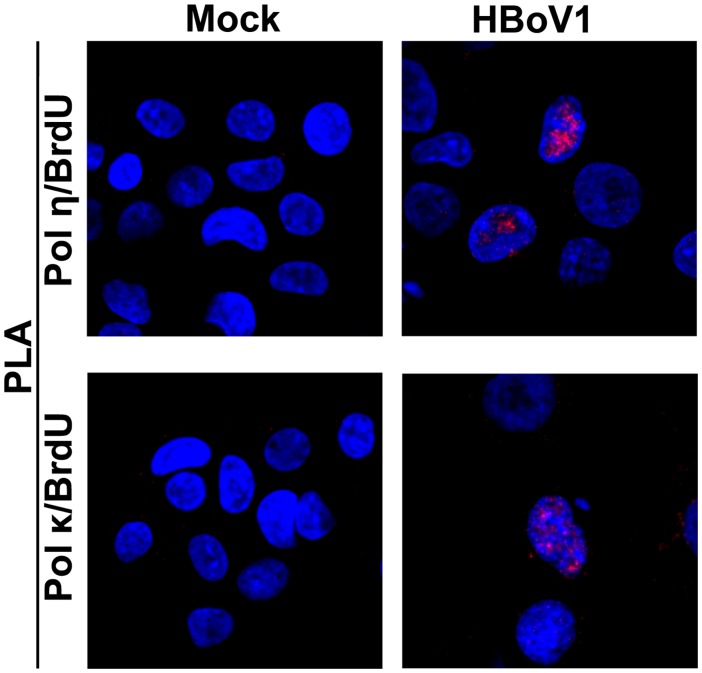
Pol η and Pol κ colocalize with the replicating HBoV1 genome. HAE-ALI cultures were infected with HBoV1 or mock-infected. At 12 dpi, infected HAE cells were trypsinized off the Transwell insert, and labeled with BrdU. The treated cells were then cytospun onto a slide, and were co-stained with a mouse anti-BrdU and a rabbit anti-Pol η antibody, or with a mouse anti-BrdU and a rabbit anti-Pol κ antibody. Proximity ligation assay (PLA) was performed following the manufacturer’s instructions. Amplified signals were visualized by confocal microscopy at a magnification of ×100.

Parvovirus DNA replication follows a rolling-hairpin model of DNA replication, in which, DNA replication factors, i.e., PCNA, RPA32 and Pol δ, are required [40,41]. However, the key DNA replication factors PCNA and Pol δ are not expressed in non-dividing HAE-ALI, as determined by IF analysis and Western blotting (S1A and S1C Fig, Fig 7A and S4 Fig). Similarly, primase Pol α and the leading strand synthesis Pol ε are also not expressed in HAE-ALI, as determined by IF analysis (Fig 7B and 7C) and Western blotting (S4 Fig). Thus, we hypothesized that the DNA polymerases utilized in DNA repair might be involved in HBoV1 DNA replication within non-dividing airway epithelial cells. We next examined the Y-family DNA repair polymerase Pol η, Pol ι and Pol κ, B-family polymerase DNA Pol ζ and Pol Rev 1, and the X-family polymerase Pol β, Pol λ, and Pol μ, which are important DNA polymerases in DNA repair [42]. Notably, Pol η and Pol κ were expressed in non-dividing HAE cells of the ALI cultures (Fig 7D and 7F), while Pol ι (Fig 7E), Pol Rev1 and Pol ζ (Fig 7G and 7H), Pol β, Pol μ, and Pol λ (Fig 8) were not, as determined by IF analysis and also confirmed by Western blotting (S4 Fig). We next visualized interactions between Pol η and Pol κ with nascent replicating viral DNA that was pulse-labeled with BrdU in HBoV1-infected cells using a proximity ligation assay (PLA). We observed clearly positive fluorescent foci in HBoV1-infected HAE cells stained with anti-Pol η and anti-BrdU antibodies, as well as with anti-Pol κ and anti-BrdU antibodies, but not in mock-infected cells (Fig 9), suggesting a direct interaction of Pol η and Pol κ with the replicating viral genomes.

**Fig 10:**
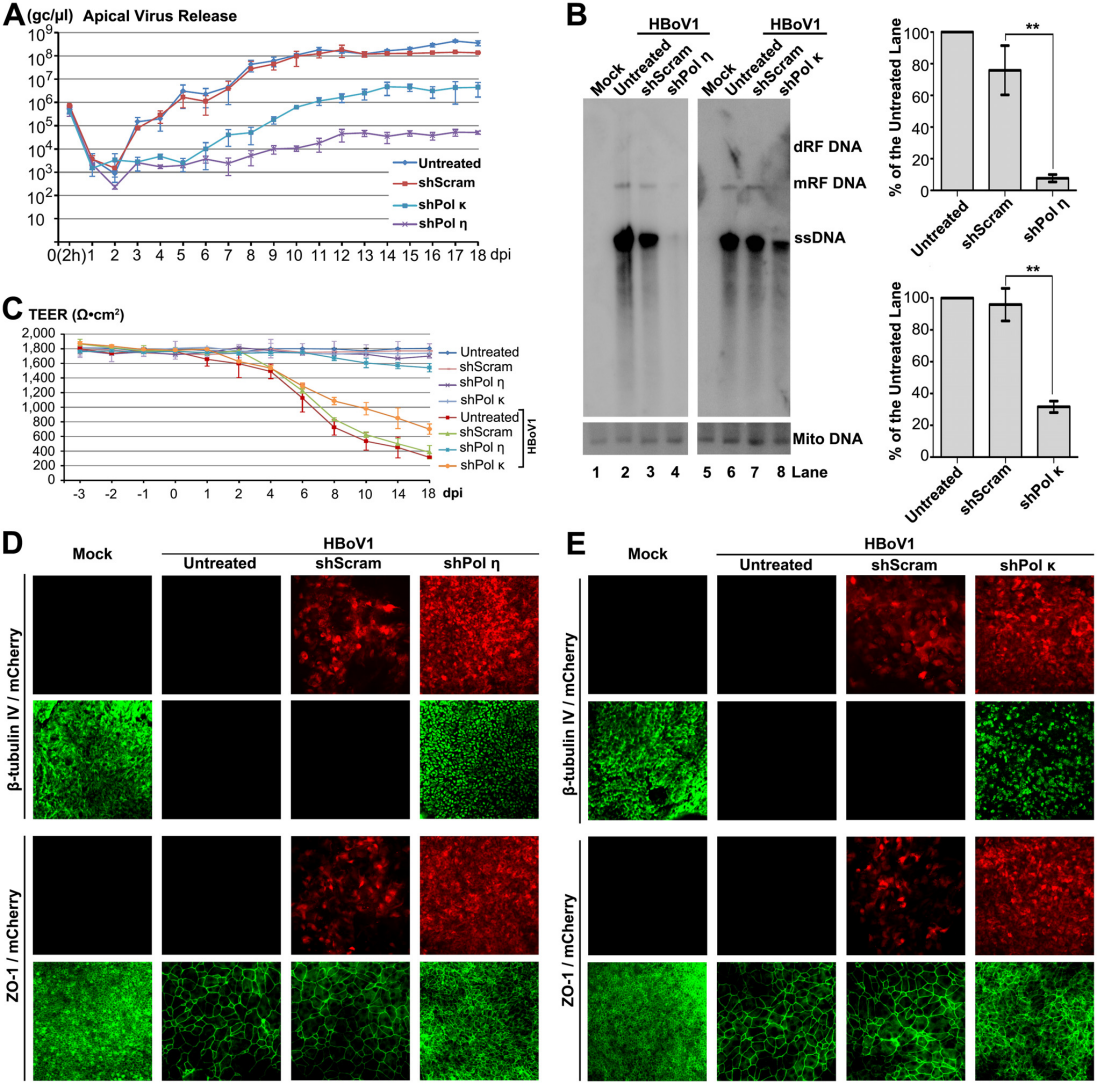
Pol η- and Pol κ-specific shRNAs inhibit HBoV1 DNA replication and attenuate the epithelial damage caused by HBoV1 infection. The HAE-ALI cultures either untreated or treated with shScram, shPol η, and shPol κ, as indicated, were infected with HBoV1. (**A**) Quantification of apical virus release. At the indicated dpi, daily apical washes were quantified for viral genome copies, which are plotted to the dpi as shown. Means and standard deviations (n = 3) are shown. (**B**) Southern blot analysis of viral DNA replication. At 18 dpi, Hirt DNA samples were extracted from infected cultures, and analyzed for viral DNA (upper) by Southern blot. Mito DNA was probed as a recovery control. A representative blot is shown. The levels of viral ssDNA in the blot were quantified and normalized to the level of Mito DNA in the same sample. The % of the viral ssDNA relative to that of the “Untreated” sample is shown. Averages and standard deviations (n = 3) were shown to the right. P values are calculated using a Student’s “*t*” test (** P<0.05). N.S. (P>0.1) indicates no statistically significant difference. (**C**) TEER measurement. At the indicated dpi, the TEER of infected HAE-ALI cultures, which expressed various shRNAs, as indicated, was measured. Means and standard deviations (n = 3) are shown. (**D** and **E**) IF analysis. At 18 dpi, infected HAE-ALI was stained with anti-β-tubulin IV or with anti-ZO-1. The stained membranes were visualized for β-tubulin IV/ZO-1 (green) and mCherry (red) expression by confocal microscopy at a magnification of × 40.

We then sought to knock down Pol η and Pol κ and directly interrogate their functions in HBoV1 genome amplification in HAE-ALI, using lentiviral vectors that expressed Pol η- and Pol κ-specific shRNAs (shPol η- and shPol κ). The lentiviral vector transduction and polarization of the transduced airway cells were conducted in the same manner as the PI3KK shRNA study described above. After HBoV1 infection, the shPol η-expressing HAE-ALI had decreases in apical virion release of 2–3 log and >3 log at 4–8 dpi and 9–18 dpi, respectively; while shPol κ-expressing HAE-ALI showed a decrease of 1 log at 3–4 dpi and of >2 log at 5–18 dpi in apical virus release, compared with the shScram-expressing HAE-ALI (Fig 10A). At 18 dpi, Southern blot analysis of viral DNA replication showed that there was 10-fold and 3-fold reductions in the level of viral ssDNA in shPol η- and shPol κ-expressing cells, respectively, compared with the shScram controls (Fig 10B). Western blotting showed that shPol η and shPol κ knocked down Pol η- and Pol κ, respectively, by 3.2-fold and 2.5-fold in HAE-ALI (S3C and S3D Fig). In infected HAE-ALI, shPol η-expressing HAE-ALI demonstrated protection from HBoV1 infection-dependent decrease in TEER, while shPol κ and shScram-expressing HAE-ALI did not (Fig 10C). However, both shPol η and shPol κ protected the infected HAE from HBoV1 infection-dependent loss of cilia (β-tubulin IV expression) and dissociation of the tight junction protein ZO-1, to various extents, compared to the shScram-controls (Fig 10D and 10E). The expression of shRNAs alone did not have an obviously deleterious effect on the HAE-ALI, as indicated by the TEER (Fig 10C).

Taken together, our results provide evidence that the DNA repair polymerases Pol η- and Pol κ are involved in HBoV1 genome amplification in non-dividing HAE cells.

## Discussion

Autonomous parvovirus DNA replication is thought in general to rely on the activity of host DNA replication machinery of the cells at S phase of the cell cycle during cell proliferation. However, we demonstrate for the first time that genome amplification of a member of autonomous parvovirus occurs in non-dividing cells. We confirmed that productive infection of HBoV1 in non-dividing airway epithelial cells employs the cellular DNA damage and repair machinery to amplify the viral genome. This innovative finding solves the puzzle of how HBoV1 amplifies its genome in terminally differentiated airway epithelial cells and causes structural lesions in the airway. Notably, all three PI3KKs (ATM, ATR, and DNA-PKcs) are phosphorylated at sites (ATM at serine1981, ATR at threonine1989, and DNA-PKcs serine 2056) that are functionally required to transduce DDR signaling [33–35]. Importantly, we found clues that the Y-family DNA polymerases Pol η and Pol κ play a role in HBoV1 genome amplification. Thus, our study provides direct evidence that the concomitant DDR induced by virus infection recruits cellular DNA repair polymerases, which can be utilized for viral genome amplification.

The group of small DNA viruses, including dsDNA papillomavirus and polyomavirus, and ssDNA parvovirus and circovirus, do not encode viral DNA polymerase, and, therefore, they must employ host DNA polymerases for their genome amplification. Most of these small DNA viruses use a strategy of either replicating in dividing cells [40] or inducing infected cells to enter the S phase of the cell cycle by expressing an oncogenic viral protein [43]. There are only a few exceptions of small DNA viruses that replicate in differentiated cells, e.g., human papillomaviruses (HPV). HPV productive infection is tightly associated with epithelial differentiation [44], and its active genome amplification is dependent on the activation of the ATM-mediated DNA repair pathway [45,46]. However, it remains unclear how HPV employs the DNA repair machinery for viral genome amplification in differentiated epithelial cells [44].

In dividing cells, infection of autonomous parvoviruses induces a DDR with at least one of the PI3KKs activated [47–52]. Activation of ATM is critical to the replication of the *Protoparvovirus* MVM [49,50] and *Bocaparvovirus* minute virus of canines (MVC) [47]; whereas activation of ATR and DNA-PKcs plays a key role in *Erythroparvovirus* B19 DNA replication [48]. Notably, an apparent cell cycle arrest at S or late S/G2 phase is always accompanied with the DDR induced by infections of these parvoviruses [28,51,53]. However, on the other hand, autonomous parvovirus replication relies on the host cell DNA replication machinery, and is dependent on the S phase of the infected cells [19–22,51]. Therefore, the DDR-facilitating parvovirus DNA replication during infections of MVM, MVC, and B19 is likely a result of cell cycle arrest at the S/late S phase induced by ATM or ATR activation. As for *Dependoparvovirus*, AAV2 infection in the presence of helper virus induces activation of DNA-PKcs and ATM [54,55]. Without helper viruses and any AAV2 protein expression, the infection of UV-inactivated AAV2 infection activates ATR by mimicking a stalled replication fork, and induces G2/M arrest and apoptosis [56]. Unfortunately, all these studies were performed in dividing cells. Of note, recombinant AAV (rAAV) vector transduces non-dividing cells efficiently [57], which is one of the advantages in using rAAV vector in human gene therapy [41]. DNA-damaging agents have been reported to greatly increase the transduction of non-dividing cells by rAAV [58]. Nevertheless, how the ssDNA genome of rAAV converts to transcription-capable dsDNA form in non-dividing cells is still elusive. Additionally, AAV replication can also be stimulated in the absence of helper viruses by treatments that cause cellular genotoxic stress [59]. These agents include hydroxyurea, topoisomerase inhibitors, and UV irradiation. Exactly how these treatments create a favorable environment for AAV replication remains unclear.

The involvement of the Y-family DNA polymerase Pol η and Pol κ in HBoV1 genome amplification suggests a DNA repair model of HBoV1 genome amplification. The HBoV1 genome contains heterogeneous terminal repeats at two ends, a DNA molecule similar to ssDNA break that normally induces ATR activation [60]. The hairpinned HBoV1 genome may be recognized by ATR and repaired by the Y-family polymerases following a DNA repair mechanism. DNA polymerase Pol η is a eukaryotic DNA polymerase involved in the DNA repair by translesion synthesis (TLS) [42]. It is particularly important in allowing accurate translesion synthesis of DNA damage resulting from ultraviolet radiation, and in some cases, Pol η can perform DNA repair at high fidelity [61]. DNA polymerase Pol κ that is specifically involved in DNA repair, also plays an important role in translesion synthesis, where the normal high-fidelity DNA polymerases cannot proceed and DNA synthesis stalls [62]. The translesion DNA synthesis by the Y-family DNA polymerases is mediated via interaction with mono-ubiquitination of PCNA [63]. However, that fact that PCNA is not expressed in non-dividing airway epithelial cells suggests that HBoV1 genome amplification follows a PCNA-independent DNA repair pathway. The activation of ATM and the importance of its signaling suggest that the homologous recombinational repair (HRR) pathway plays a role in HBoV1 genome amplification of differentiated epithelial cells. The high fidelity HRR has been implicated in HPV late gene amplification in differentiated epithelial cells [46,64].

Studies have revealed that the majority of the rAAV vector genomes persist as circular episomes of monomers or concatemers in tissues [65,66], and have identified host DNA repair factors in ATM and DNA-PK pathways involved in processing AAV genomes in non-dividing cells [67,68]. In addition, during replication of the *Protoparvovirus* MVM, the 3’ end of the newly synthesized complementary strand is ligated to the right-end hairpin of the viral genome, resulting in the formation of a covalently closed RF DNA, which is the major conversion product [69]. It is impossible that this ligation is carried out by the DNA-PK complex. In fact, DNA-PK has been proved to play a role in AAV replication using both in vivo and in vitro replication assays [70].

The detailed mechanism underlying how ATR, ATM, and DNA-PKcs, functioning either independently or synergistically, mediate HBoV1 genome amplification in non-dividing cells, especially the involvement of the DNA repairing factors, warrants further investigation. Although AAV2 is a *Dependoparvovirus*, it was observed that the AAV2 genome replicates autonomously in a skin raft model of differentiated keratinocytes [71]. We believe that a differentiation (DNA repair)-dependent viral DNA replication probably exists as a general mechanism of parvovirus DNA replication in non-dividing cells. We find HBoV1 infects only cells of polarized primary airway epithelium, and thus, we could not examine the DDR-supported viral DNA replication in other types of non-dividing cells. The non-dividing HAE infection model is essential in understanding the mechanism underlying the role of parvovirus infection-induced DDR signal transduction in facilitating viral DNA synthesis, without disturbing the cell cycle as in dividing cells. We speculate that it is highly likely that other small DNA viruses, in addition to HBoV1, utilize cellular DNA repair factors, in particular the Y-family DNA polymerases, for their genome amplification in non-dividing cells. Viruses have evolved in a way to utilize various host DNA polymerases depending on which ones are available in the host cells. A concrete understanding of these pathways may enhance the development of anti-viral therapies and also may improve the utility of recombinant vectors that utilizes these viruses for gene therapy.

## Materials and Methods

### Ethics statement

Primary human airway (tracheobronchial) epithelial cells were isolated from the lungs of healthy human donors at Cell Culture Core of the Center for Gene Therapy, University of Iowa, under IRB approval by the Institutional Review Board of the University of Iowa (IRB ID No. 9507432). We obtained the well differentiated (polarized) human airway epithelium (HAE) ALI cultures from at the Cell Culture Core without any identification information on them, and, therefore, an IRB review was waived.

### Human airway cell culture and ALI differentiation

Primary human airway (tracheobronchial) epithelial cells were cultured on collagen-coated, semipermeable membrane inserts (0.6 cm^2^, Millicell-PCF; EMD-Millipore, Billerica, MA; or 0.33 cm^2^, Transwell, Corning, Tewksbury, MA), and then were differentiated at an ALI for 3–4 weeks [16]. This procedure was carried out at the Tissue and Cell Culture Core of the Center for Gene Therapy, University of Iowa. In some circumstances, primary human airway epithelial cells of HAE-ALI cultures were propagated within a fibroblast feeder cell system in F medium, in which cells were co-cultivated with irradiated 3T3 fibroblast (J2 strain) with the addition of ROCK inhibitor Y-276322 [15,72], and then were transferred into a Transwell insert (0.33 cm^2^, Transwell) for ALI differentiation. Briefly, 2×10^4^ airway cells were seeded onto the Transwell insert. In the first 2 to 3 days, F medium was fed in both the apical and basolateral chambers of the insert. Then, F medium was aspirated from both chambers, and the cells were fed only with 500 μl of PneumaCult-ALI medium (StemCell, Vancouver, BC, Canada) in the basolateral chamber. The medium was changed every 3–4 days, and the ALI-cultured HAE took 3–4 weeks for full differentiation. We chose the cultures with a transepithelial electrical resistance (TEER) of over 1,500 Ω∙cm^2^, as determined with an epithelial Ohm-voltmeter (Millicell-ERS; EMD-Millipore), for subsequent HBoV1 infection.

### Virus, infection, and quantification of apical virus release

HBoV1 virions were collected from apical washes of the HBoV1-infected HAE-ALI and were used for infection at a multiplicity of infection (MOI) of 1 viral genome copy number (gc)/cell, as described previously [17].

At various time points, 100 μl aliquots of phosphate buffered saline, pH7.4 (PBS) were added to the apical chamber of the HAE-ALI culture, and were harvested as apical washes. All the washes were stored at 4°C for quantification of viral genome copy numbers using a quantitative PCR (qPCR) with HBoV1-specific primers and probe, essentially following the method described previously [16].

### Chemicals and treatments

Hydroxyurea (HU; Calbiochem, EMD Millipore) was dissolved in deionized water to make a 200 mM stock solution. The following pharmacological inhibitors were used in this study: KU60019 (an ATM-specific inhibitor, Tocris Bioscience, Bristol, UK), AZ20 (an ATR-specific inhibitor, Selleckchem, Houston, TX), and NU7441 (a DNA-PKcs-specific inhibitor, Tocris Bioscience). All inhibitors were dissolved in dimethyl sulfoxide (DMSO) to make stock solutions at 10 mM.

Inhibitors were applied 2 days prior to infection, and were included in the ALI medium throughout the experimental period, which was refreshed every three days.

### Immunofluorescence (IF) analysis

Differentiated airway epithelial cells on the ALI membrane support of the HAE-ALI cultures were treated with 0.05% trypsin for 5 min, washed once with PBS, and collected in 200 μl of PBS (~5 × 10^4^ cells). The proliferating primary human airway epithelial cells or the differentiated airway epithelial cells isolated from the ALI membrane were cytospun onto a slide at 2,000 rpm for 5 min. The cells were then air-dried for 1 hr at room temperature. For analysis of β-tubulin IV and ZO-1 expression in differentiated cells on the ALI membrane, we directly stained the ALI culture.

IF analysis was essentially followed using a method described previously [16], with antibodies against proteins as indicated in the figures. Confocal images were taken with an Eclipse C1 Plus confocal microscope (Nikon) controlled by Nikon EZ-C1 software. DAPI (4’,6-diamidino-2-phenylindole) was used to stain the nucleus.

### BrdU incorporation assay

Differentiated airway epithelial cells were treated with 5 mM EDTA for 5 min and then trypsinized off the insert of the infected HAE-ALI. Approximately 1 × 10^5^ cells were resuspended in 1 ml of the PneumaCult-ALI medium (StemCell) with BrdU (Sigma, St Louis, MO) at a final concentration of 30 μM and incubated for 20 min. Next, cells were cytospun onto slides for IF analysis with anti-BrdU and anti-HBoV1 NS1C antibodies. For the detection of cellular DNA replication, BrdU-incorporated cells were further treated with 1 M HCl for 30 min to denature chromosome DNA [28].

### Proximity ligation assay (PLA)

PLA was performed using the Duolink PLA Kit (Sigma) according to the manufacturer’s instructions. HAE cells were collected from the Transwell insert and were labeled with BrdU as described above. At room temperature, the cells were fixed with 3.7% paraformaldehyde for 15 min, permeabilized with 0.2% Triton X-100 for 5 min, and blocked with Duolink Blocking Buffer for 30 min. Then, the cells were incubated with primary antibodies, mouse anti-BrdU and rabbit anti-Pol η or with mouse anti-BrdU and rabbit anti-Pol κ, for 1 hr. Two diluted PLA probes, which are specific to mouse and rabbit IgG, respectively, were applied to the cells and incubated for 60 min at 37°C. The hybridized oligonucleotides were ligated in the Ligation Solution at 37°C for 30 min and amplified in Amplification Solution for 100 min. Finally, the cells were washed and mounted with Duolink In Situ Mounting Medium with DAPI and visualized under a Nikon Eclipse C1 Plus confocal microscope.

### Western and Southern blot analyses

For Western blotting, the HAE cells on the insert of the ALI culture were lysed in 200 μl of 1 × SDS-loading buffer. Lysed samples were loaded for SDS-polyacrylamide gel electrophoresis (PAGE), transferred, and blotted with antibodies as indicated in the figures, as previously described [16]. Images were developed under the imager FUJIFILM LAS 4000 (FUJIFILM Life Sciences) and quantified with Multi Gauge V2.3 software (FUJIFILM Life Sciences).

For Southern blotting, HAE cells were trypsinized off the insert of the ALI culture, washed and collected for extraction of low molecular weight (Hirt) DNA [73,74]. Southern blotting was performed using an HBoV1 *NS* and *Cap* gene probe, as previously described [27]. A mitochondrial DNA probe was used as a control for the recovery of the Hirt DNA [75]. Images were developed with a Typhoon FLA 9000 phosphor imager and quantified using ImageQuant TL 8.1 (GE Healthcare).

### Lentiviral vector production and transduction of HAE-ALI cultures

pLKO-mCherry backbone vector was constructed by inserting a CMV-driven mCherry gene into pLKO.1 vector (Addgene, Inc., Cambridge, MA) through the BstB I and Mfe I sites. shRNAs sequences, which are generated by annealing oligonucleotides synthesized at Integrated DNA Technologies (IDT; Coralville, IA), were cloned into the pLKO-mCherry using the Age I and EcoR I sites. Lentiviral vectors were produced and purified as previously described [76].

To generate shRNA-expressing HAE-ALI cultures, proliferating airway epithelial cells cultured as monolayer were infected with lentiviral vector at an MOI of ~10. After 1 day, transduced cells were transferred into Transwell inserts. After 2–3 days, PneumaCult–ALI medium (StemCell) was used to establish an ALI for polarization, as described above.

### Cytotoxicity assay

Cell viability was quantified using a Cytotoxicity Assay kit (Promega, Madison, WI) following the manufacturer’s instructions. Briefly, HAE-ALI cultures were treated with KU60019 (40 μM), AZ20 (20 μM), and NU7441 (20 μM) for 23 days. Staurosporine was used as a positive control in different final concentrations (2, 20, and 200 μM) for 2 days. DMSO at 0.1% was used as a vehicle control. At the end of treatment, HAE cells were collected from the Transwell inserts and seeded into a 96-well plate, followed by addition of the cytotoxicity assay reagents. After incubations, luminescence was measured by a Synergy H1 microplate reader (BioTek U.S., Winooski, VT). Then, lysis reagent was added into the mixtures, after incubation luminescence was measured again. The dead cell numbers and total cell numbers were determined from the first luminescence and second luminescence results, respectively. The cell viabilities were normalized to the “Untreated” group.

### Antibodies used

Rat anti-HBoV1 NS1C antibody was produced previously [77]. The following antibodies were purchased: anti-p27, anti-PCNA, anti-Ki67, and anti-BrdU from BD Biosciences (San Jose, CA), anti-phosphorylated H2AX (γ-H2AX) from Millipore, anti-phosphorylated replication protein A32 (p-RPA32 on serine 33), anti-p-ATR (Thr1989), anti-Pol ι, and anti-Pol ε from GeneTex (Irvine, CA), anti-p-ATM (Ser1981), anti-p-DNA-PKcs (Ser2056), anti-ATR, anti-Pol κ, anti-Pol ζ, and anti-Pol η from Abcam (Cambridge, MA), anti-ATM from Cell Signaling Inc. (Danvers, MA), anti-DNA-PKcs from Biolegend, anti-Pol α, anti-Pol δ, anti-Rev1 and anti-Pol β from Santa Cruz (Dallas, Texas), and anti-β-actin from Sigma. An anti-Pol ι antibody from Bethyl Laboratories, Inc. (Montgomery, TX) was used for Western blotting.

### Oligonucleotides used to generate shRNA sequences

The following shRNA sequences were chosen to target the genes of interest: shRNA specific to ATM (shATM), 5’- CCG GGA TTT GCG TAT TAC TCA GTC TCG AGA CTG AGT AAT ACG CAA ATC CTT TTT G-3’ [78]; shRNA specific to ATR (shATR), 5’- CCG GGG CGT CGT CTC AGC TCG TCT CCT CGA GGA GAC GAG CTG AGA CGA CGC CTT TTT G-3’; shRNA specific to DNA-PKcs (shDNA-PKcs) [78], 5’-CCG GGA TCG CAC CTT ACT CTG TTC TCG AGA ACA GAG TAA GGT GCG ATC TTT TTG-3’ [79]; shRNA specific to Pol η (shPol η), 5’-CCG GCC CGC TAT GAT GCT CAC AAG ACT CGA GTC TTG TGA GCA TCA TAG CGG GTT TTT G-3’ [80]; shRNA specific to DNA Pol κ (shPol κ), 5’-CCG GGC CAT TGC TAA GGA ATT GCT ACT CGA GTA GCA ATT CCT TAG CAA TGG CTT TTT G-3’ (Sigma, TRCN0000115999). The following scrambled shRNA (shScram) was used as an shRNA control: 5’-CCG GCC TAA GGT TAA GTC GCC CTC GCT CGA GCG AGG GCG ACT TAA CCT TAG GTT TTT G-3’ [48].

## Supporting Information

S1 Fig Cell cycle status of the HAE-ALI cultures and HBoV1 infection of dividing cells. (**A**, **B**, and **C**) Primary airway epithelial cells of HAE-ALI cultures are well differentiated. (**A** and **B**) Immunofluorescence (IF) analysis. Both monolayer- and ALI-cultured epithelial cells, marked as “Monolayer” and “HAE-ALI,” respectively, were trypsinized, cytospun onto slides, and analyzed by IF with anti-PCNA (**A**) and anti-p27 (**B**). Nuclei were stained with DAPI (blue), and the cells were visualized by confocal microscopy at a magnification of × 60. **(C)** Western blot analysis. Both monolayer- and ALI-cultured epithelial cells, marked as “Monolayer” and “HAE-ALI,” respectively, were analyzed by Western blotting using antibodies against proteins as indicated. Each blot was reprobed for β-actin as a loading control. (**D**) HBoV1 infection of dividing cells. Monolayer-cultured (dividing) primary airway epithelial cells were used to infect HBoV1 at an MOI of ~10, or were mock-infected. At 3 dpi, infected cells were analyzed by IF with anti-NS1C and anti-p27 antibodies, and with anti-NS1C and anti-Ki67 antibodies, respectively.
(TIF)

S2 Fig Cell viability analysis of inhibitor-treated HAE-ALI cultures. HAE-ALI cultures were treated with pharmacological inhibitors, as indicated. At 23 days post-treatment, cells were harvested to assess viability based on ATP release using the Cytotoxicity Assay kit (Promega). The normalized viabilities, relative to the “Untreated” group, are plotted. Means and standard deviations (n = 3) are shown. Staurosporine was used as positive control at various concentrations but only for 2 days. N.S. (P>0.1) indicates no statistically significant difference. ***P<0.01 and ****P<0.001 (by Student’s “*t*” test).
(TIF)

S3 Fig Expression of shRNAs in HAE-ALI cultures. Proliferating primary airway epithelial cells cultured on Transwell inserts were transduced with shRNA-expressing lentiviruses, as indicated, or were untreated. One day later, the cells were cultured at an ALI. (**A**) mCherry expression. At weeks after an ALI, as indicated, the transduced cells were monitored for mCherry expression by taking images at a magnification of ×10 under an Eclipse Ti-S microscope (Nikon). (**B**) Western blot analysis of ATM, ATR, and DNA-PKcs knockdown. At 4 weeks at ALI, cells of the HAE-ALI cultures treated with shRNA, as indicated, or of untreated were analyzed for expression of ATM, ATR and DNA-PKcs, as indicated, with β-actin as a loading control by Western blotting. (**C** and **D**) Western blot analysis of Pol η and Pol κ knockdown. At 4 weeks at ALI, prior to virus infection, cells of each shRNA-transduced HAE-ALI culture were analyzed for expression of Pol η (**C**) and Pol κ (**D**), with β-actin as a loading control by Western blotting. Representative blots are shown. The bands of Pol η (C) and Pol κ (D) were quantified and normalized to the β-actin band of each lane. The % of Pol η (C) or Pol κ (D) expression level relative to that of the “Untreated” sample is shown. Averages and standard deviations (n = 3) are shown. **P<0.05, N.S.: P>0.1 (by Student’s “*t*” test).
(TIF)

S4 Fig Western blot analysis of DNA polymerases expressed in HAE-ALI. Monolayer (Dividing)- or ALI (Non-dividing)-cultured epithelial cells were collected at equivalent numbers and lysed for Western blotting using antibodies against DNA polymerases Pol δ, Pol α, Pol ε, Pol η, Pol ι, Pol κ, Pol Rev1, Pol ζ, Pol β, Pol μ, and Pol λ, as indicated. β-actin was detected as a loading control.
(TIF)
