# Supplementary figures and images for: Replication of an Autonomous Human Parvovirus in Non-dividing Human Airway Epithelium Is Facilitated through the DNA Damage and Repair Pathways
Source: PLoS Pathog. 2016 Jan 14;12(1):e1005399. doi: 10.1371/journal.ppat.1005399 (PMC4713420; doi:10.1371/journal.ppat.1005399)

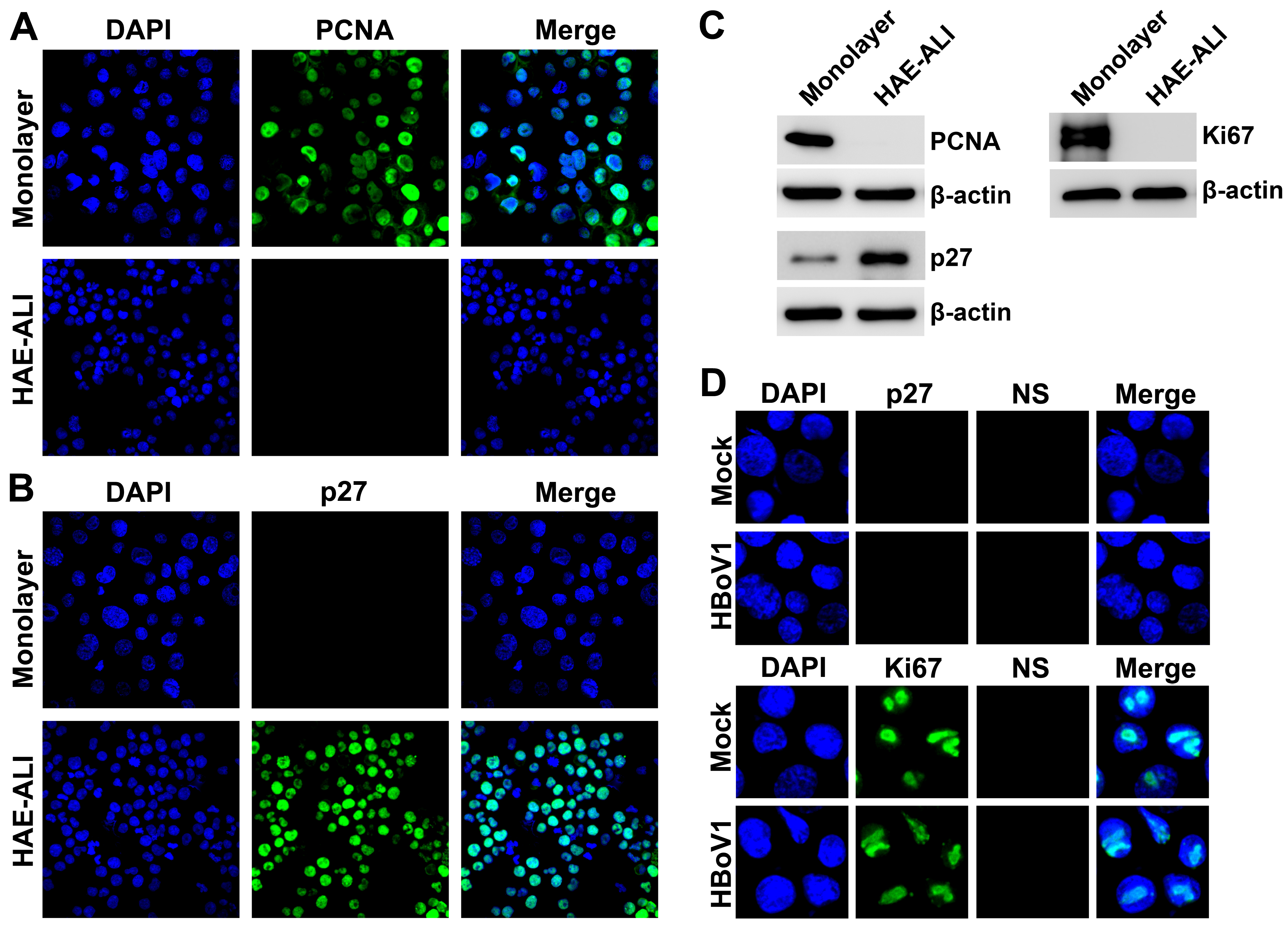

Supplement: S1 Fig — (A, B, and C) Primary airway epithelial cells of HAE-ALI cultures are well differentiated. (A and B) Immunofluorescence (IF) analysis. Both monolayer- and ALI-cultured epithelial cells, marked as “Monolayer” and “HAE-ALI,” respectively, were trypsinized, cytospun onto slides, and analyzed by IF with anti-PCNA (A) and anti-p27 (B). Nuclei were stained with DAPI (blue), and the cells were visualized by confocal microscopy at a magnification of × 60. (C) Western blot analysis. Both monolayer- and ALI-cultured epithelial cells, marked as “Monolayer” and “HAE-ALI,” respectively, were analyzed by Western blotting using antibodies against proteins as indicated. Each blot was reprobed for β-actin as a loading control. (D) HBoV1 infection of dividing cells. Monolayer-cultured (dividing) primary airway epithelial cells were used to infect HBoV1 at an MOI of ~10, or were mock-infected. At 3 dpi, infected cells were analyzed by IF with anti-NS1C and anti-p27 antibodies, and with anti-NS1C and anti-Ki67 antibodies, respectively. (TIF) [file ppat.1005399.s001.tif]

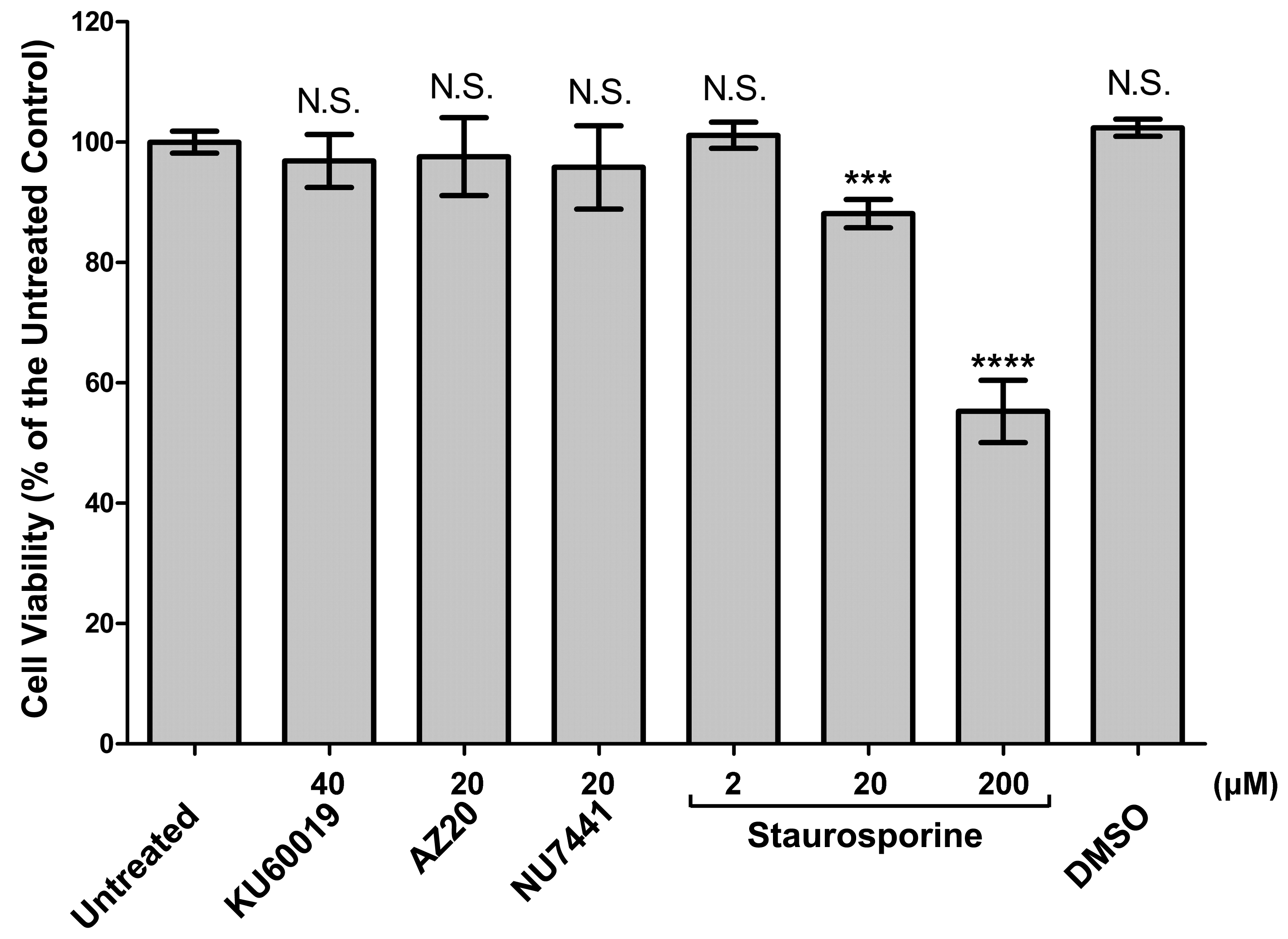

Supplement: S2 Fig — HAE-ALI cultures were treated with pharmacological inhibitors, as indicated. At 23 days post-treatment, cells were harvested to assess viability based on ATP release using the Cytotoxicity Assay kit (Promega). The normalized viabilities, relative to the “Untreated” group, are plotted. Means and standard deviations (n = 3) are shown. Staurosporine was used as positive control at various concentrations but only for 2 days. N.S. (P>0.1) indicates no statistically significant difference. ***P<0.01 and ****P<0.001 (by Student’s “t” test). (TIF) [file ppat.1005399.s002.tif]

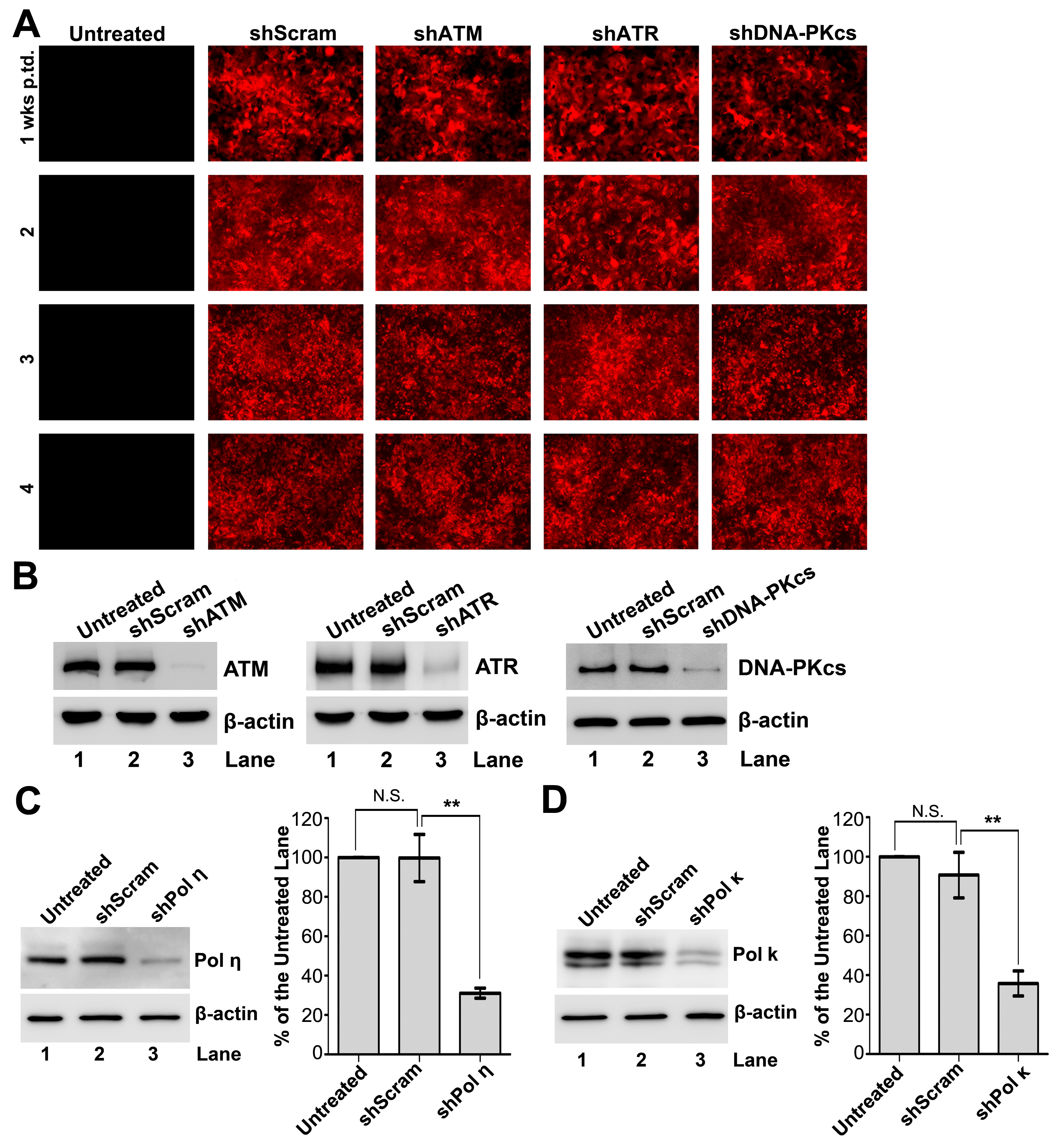

Supplement: S3 Fig — Proliferating primary airway epithelial cells cultured on Transwell inserts were transduced with shRNA-expressing lentiviruses, as indicated, or were untreated. One day later, the cells were cultured at an ALI. (A) mCherry expression. At weeks after an ALI, as indicated, the transduced cells were monitored for mCherry expression by taking images at a magnification of ×10 under an Eclipse Ti-S microscope (Nikon). (B) Western blot analysis of ATM, ATR, and DNA-PKcs knockdown. At 4 weeks at ALI, cells of the HAE-ALI cultures treated with shRNA, as indicated, or of untreated were analyzed for expression of ATM, ATR and DNA-PKcs, as indicated, with β-actin as a loading control by Western blotting. (C and D) Western blot analysis of Pol η and Pol κ knockdown. At 4 weeks at ALI, prior to virus infection, cells of each shRNA-transduced HAE-ALI culture were analyzed for expression of Pol η (C) and Pol κ (D), with β-actin as a loading control by Western blotting. Representative blots are shown. The bands of Pol η (C) and Pol κ (D) were quantified and normalized to the β-actin band of each lane. The % of Pol η (C) or Pol κ (D) expression level relative to that of the “Untreated” sample is shown. Averages and standard deviations (n = 3) are shown. **P<0.05, N.S.: P>0.1 (by Student’s “t” test). (TIF) [file ppat.1005399.s003.tif]

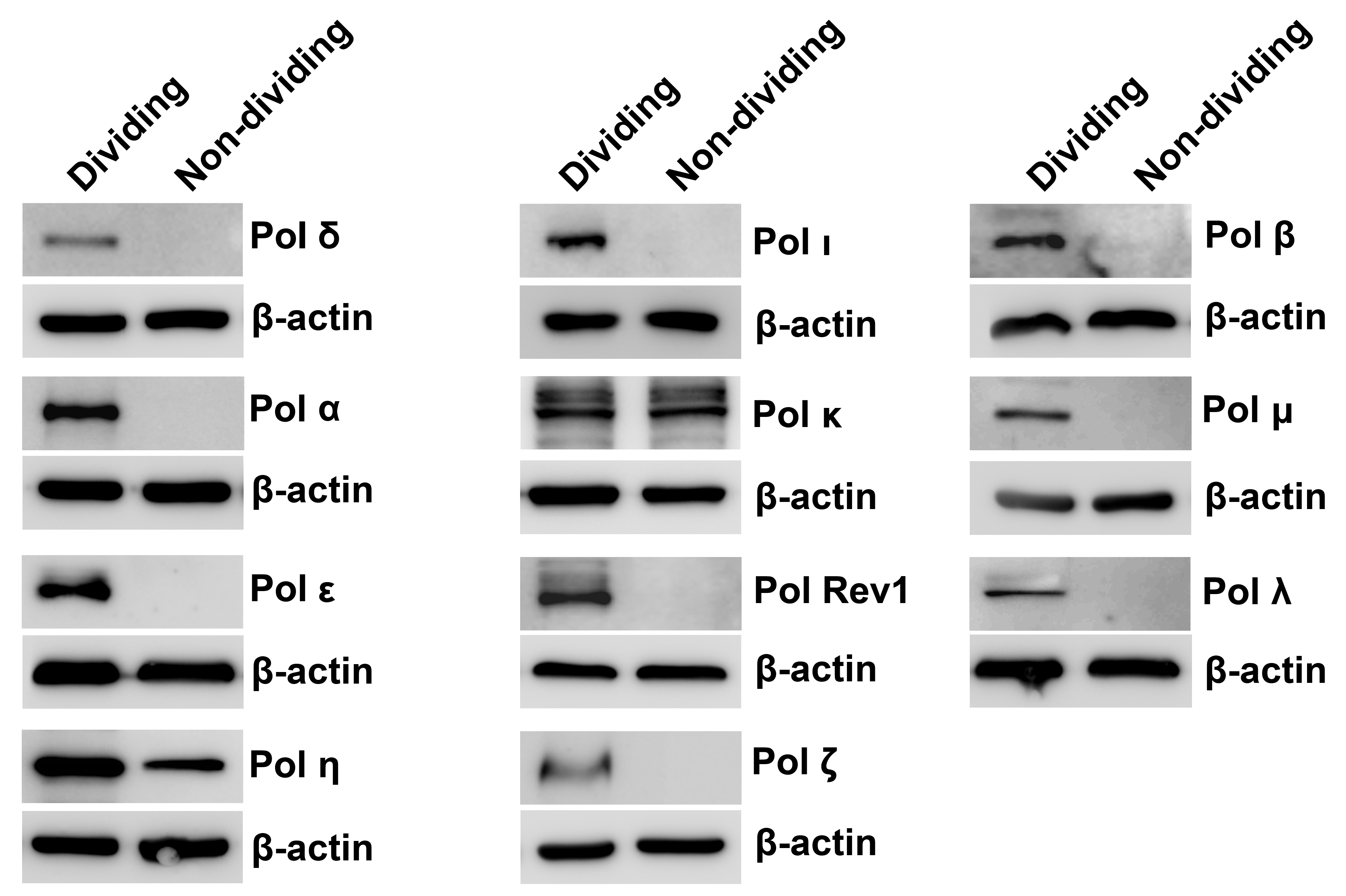

Supplement: S4 Fig — Monolayer (Dividing)- or ALI (Non-dividing)-cultured epithelial cells were collected at equivalent numbers and lysed for Western blotting using antibodies against DNA polymerases Pol δ, Pol α, Pol ε, Pol η, Pol ι, Pol κ, Pol Rev1, Pol ζ, Pol β, Pol μ, and Pol λ, as indicated. β-actin was detected as a loading control. (TIF) [file ppat.1005399.s004.tif]
